# Polysaccharides from Radix *Peucedani*: Extraction, Structural Characterization and Antioxidant Activity

**DOI:** 10.3390/molecules28237845

**Published:** 2023-11-29

**Authors:** Jie Zhang, Chenyue Wang, Qian Li, Wei Liang

**Affiliations:** State Key Laboratory of Aridland Crop Science, College of Agronomy, Gansu Agricultural University, Lanzhou 730070, China; jie.zhang101@icloud.com (J.Z.); wcy04042023@163.com (C.W.); liangw@gsau.edu.cn (W.L.)

**Keywords:** Radix *Peucedani*, polysaccharides, deep eutectic solvents, network pharmacology, molecular docking, antioxidant

## Abstract

In this study, an ultrasound-assisted green extraction method was applied for the extraction of polysaccharides from Radix *Peucedani* based on deep eutectic solvents (DESs), and the result showed that a DES system composed of betaine and 1,2-propylene glycol with a molar ratio of 1:2 possessed the optimal extraction efficiency for polysaccharides. Single-factor and Box–Behnken designs were used to determine the optimum extraction conditions for the maximum yields of polysaccharides from Radix *Peucedani* by using DESs. The maximum yields of polysaccharides attained 11.372% within a DES water content of 19%, an extraction time of 36 min, an extraction temperature of 54 °C, a solid–liquid ratio of 1:30 and an ultrasonic irradiation power of 420 W. The physicochemical properties of polysaccharides were analyzed using ICS and FT-IR, and the structure morphology was observed by SEM. The polysaccharides extracted from Radix *Peucedani* exhibited general antioxidant activities in vitro including DPPH, Hydroxyl and ABTS+ radical-scavenging activity. The antioxidant mechanism of Radix *Peucedani* polysaccharides was investigated using network pharmacology and molecular docking methods. The result showed that the high binding activity of glucose and IL1B, galactose and CASP3 was recognized as a potential mechanism for the antioxidant effects of Radix *Peucedani* polysaccharides.

## 1. Introduction

Radix *Peucedani* is a commonly employed traditional Chinese medicine derived from the dried roots of *Peucedanum praeruptorum* Dunn, a perennial herbaceous plant belonging to the Umbelliferae family that is primarily distributed in southern and northeastern China [1]. Several Radix *Peucedani* preparations are routinely used in clinical practice, serving as multi-component, multi-target, and multi-pathway agents that work by modulating targets and pathways related to viral infection, immunity and inflammation, with applications such as the prevention and treatment of COVID-19 [2]. As one of the key active ingredients in traditional Chinese medicines, polysaccharides play an important role in disease treatment. Studies have demonstrated that polysaccharides exhibit a variety of biological activities, including antiviral activity against both RNA and DNA viruses [3], anti-tumor activity [4,5] and antiproliferative and proapoptotic activity in the HS-Sultan human lymphoma cell line [6]. Extraction is the first step and an essential step for the characterization and utilization of bioactive polysaccharides [7].

Water extraction based on the typical solubility characteristics of polysaccharides is extensively utilized for the separation and isolation of plant polysaccharides. However, this method is energy intensive and difficult to apply on a large scale [7]. In addition, the obtained polysaccharide products often contain large amounts of proteins, nucleic acids, pigments and other impurities, which makes downstream purification complex and time consuming. Deep eutectic solvents (DESs) are an increasingly valued class of blended solvents, composed of ecologically sustainable and environmentally friendly components featuring hydrogen-bond donor and acceptor functionalities that result in the formation of an extended hydrogen-bonding network [8]. Owing to their biocompatibility, low toxicity and various advantageous chemical properties, such as high thermal stability [9], high conductivity, low volatility, lack of flammability and non-reactivity with water, DESs are considered a sustainable option for extracting natural products from plants in various contexts. Moreover, they hold the potential to replace volatile organic solvents and ionic liquids [10]. Several reviews have highlighted the intriguing physicochemical properties of DESs and their applications in the fields of extraction [11], organic synthesis [12], nanomaterials [13] and sustainable pulping technology [14]. Meanwhile, ultrasound irradiation has emerged as an environmentally friendly and efficient extraction method that disrupts cell walls and promotes mass transfer of the cell contents through its mechanochemical effects. In particular, ultrasound has been reported to accelerate the extraction of active ingredients from plant materials at room temperature [15]. The coupling of DESs with ultrasound irradiation has resulted in remarkable success, not only enhancing the extraction efficiency but also minimizing the solution loss and simplifying the downstream separation steps for recovering the extracted substances. Previous studies have demonstrated the effective extraction of various plant components, including anthocyanins [16], flavonoids [17], dietary fiber [18] and other active ingredients [19,20].

The combination of network pharmacology and molecular docking is becoming increasingly standardized and more widely utilized for exploring the complex mechanisms of action of traditional Chinese medicines [21]. By means of public database collections or chemical identification, network pharmacology aims to clarify the chemical substance basis of traditional Chinese medicines, potential targets involved in diseases and corresponding metabolic pathways to sequentially unfold the multi-component, multi-target and multi-pathway biological characteristics of traditional Chinese medicines [22]. Molecular docking technology assesses the affinity between the core targets and core components revealed by network pharmacology research, thus verifying the accuracy of the obtained results [23]. The combination of these two methods has become a systematic strategy for studying the complex mechanisms of action of traditional Chinese medicines [24].

In this study, the efficiencies of different combinations of DESs for the extraction of polysaccharides from Radix *Peucedani* were evaluated and compared with the conventional method. After a preliminary screening, the optimal DES system was selected. Subsequently, the main extraction parameters governing the polysaccharide yield were optimized on the basis of single-factor experiments and the resulting bioactivities were assayed. Finally, network pharmacology and molecular docking techniques were utilized to investigate the mechanism underlying the antioxidant effects of the polysaccharide fractions of Radix *Peucedani*, with a view to providing a robust experimental basis for further studies of this traditional Chinese medicine.

## 2. Results and Discussion

### 2.1. Optimization of Ultrasound-Assisted Extraction of Polysaccharides Using DESs

#### 2.1.1. Influence of the DES System

**Figure 1:**
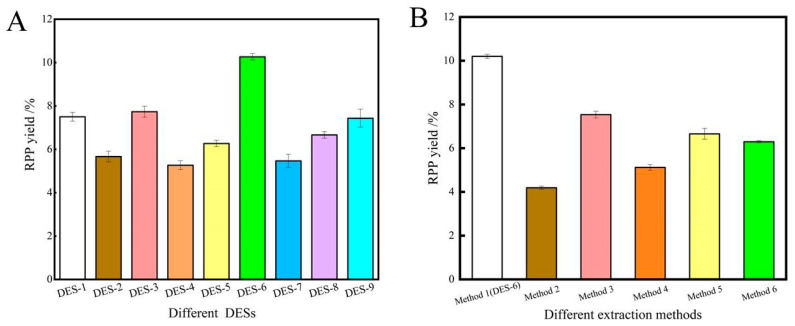
The extraction results for polysaccharides using different DESs (**A**); the extraction results for polysaccharides using different methods (**B**).

To identify the optimal extraction solvent, the polysaccharide extraction performances of nine different types of DESs and six different extraction methods were evaluated, as shown in Figure 1. The results revealed that alcohol-based DESs were the most efficient extractants, followed by organic-acid based DESs and urea-based DESs. In particular, DES-6 exhibited the highest polysaccharide extraction rate. This was ascribed to the internal space of 1,2-propanediol being sufficiently large with fewer alcohol group branches, where the strong hydrogen bonding, polysaccharides interactions and polarity were more suitable for polysaccharide extraction. Compared with traditional solvents, DESs exhibit greater binding affinity with polysaccharides due to van der Waals, hydrogen-bonding and electrostatic interactions [9]. In addition, the rupturing of the cell walls by the ultrasonic irradiation was favorable for polysaccharide solubilization [25]. Consequently, the ultrasound-assisted DES extraction method afforded the highest polysaccharide yield. On the basis of these results, DES-6 comprising betaine and 1,2-propanediol (molar ratio 1:2) was selected as the most promising solvent for the extraction of polysaccharides in subsequent experiments.

#### 2.1.2. Influence of DES Water Content on Extraction Yield

According to previous studies [26], DESs have a wide range of viscosities, which can affect the extraction efficiency. Because of the high viscosity of DES-6, DES systems with an appropriate water content facilitate mass transport. Therefore, DES-6 containing various water contents (10%, 20%, 30%, 40% and 50% *w*/*w*) was used to extract the polysaccharides. As anticipated, the extraction rate was found to be highly dependent on the water content, as shown in Table S1 (Supplementary Materials). Specifically, when the water content in the DES system was increased from 10% to 20%, the polysaccharide yield increased significantly. However, further increasing the water content (20–50%) led to a decrease in the extraction efficiency, which may be attributable to the high concentration of water limiting the reaction between the polysaccharides and the DES [10]. Therefore, a water content of 20% was considered optimal and used in subsequent experiments.

#### 2.1.3. Influence of Other Factors on Extraction Yield

The RPP were sequentially extracted under various combinations of four key parameters (extraction time, extraction temperature, ultrasonic power and material:liquid ratio) while keeping the other extraction conditions constant. The effects of these parameters on the extraction efficiency are shown in Table S1 (Supplementary Materials).

#### 2.1.4. Optimization of Extraction Conditions by Box–Behnken Design

The entire study consisted of 17 separate experiments, with each treatment being repeated three times. The factors and responses associated with this experiment are shown in Table S2 (Supplementary Materials). The regression coefficients for the intercept, linear, quadratic and interaction terms of the predictive model are listed in Table 1.

Based on the multiple regression analysis of all the experimental data, the predicted modal sentence was expressed as a second-order polynomial equation. Based on the level of coding, the regression modeling equations for responses and variables (adjusted R^2^ = 0.9597, predicted R^2^ = 0.7902) were as follows.

RBP yield(%) = 11.09 − 0.6377A + 0.9082B + 0.3879C + 0.731AB + 0.772AC − 0.8334BC − 1.42A^2^ − 0.7937B^2^ − 0.8934C^2^

where A is the content of water in DES; B is the extraction time and C is extraction temperature.

A statistically significant predictive model was demonstrated by a low *p*-value (<0.0001). Since there is no statistically significant lack of a well-fitting model (*p* > 0.05), the experimental data are accurately reflected by the predictive model. The premise that the prediction model adequately captures the experimental data is likewise supported by this finding. The relationship between the factors and the extraction rate outcomes was also made clearer by this experiment. Based on the size of the F-value, the factors influencing the RPP extraction rate were arranged in the following order: B > A > C.

#### 2.1.5. Optimization and Validation Experiments

Based on the analysis of the results of the response surface experiment, the water content of DES was 18.97%, the extraction time was 35.75 min, the extraction temperature was 54.04 °C, the extraction power was 420W and the material–liquid ratio was 1:30. Under this condition, the predicted value of the polysaccharide extraction rate was 11.365%. Considering the practical operation, the experimental conditions were set as follows: DES water content was 19%, extraction time was 36 min, extraction temperature was 54 °C, extraction power was 420 W and the material–liquid ratio was 1:30. The three-time average extraction rate of polysaccharides was 11.372%, which caused less error than the predicted value, and the model was reliable. As demonstrated in the present work, DESs can effectively extract polysaccharides from Radix *Peucedani*, which is important for the separation and isolation of bioactives and provides a new avenue for the development of polysaccharides from Radix *Peucedani*. However, considering the complexity of the bioactive compounds in many traditional Chinese medicines, the application potential of DESs for the extraction of bioactive compounds from these medicines, the optimization of the extraction processes and the technical feasibility, economy and cost of this technology still require further validation to support future industrial-scale production.

#### 2.1.6. Effect of Response Surface Interaction on Extraction Rate

**Figure 2:**
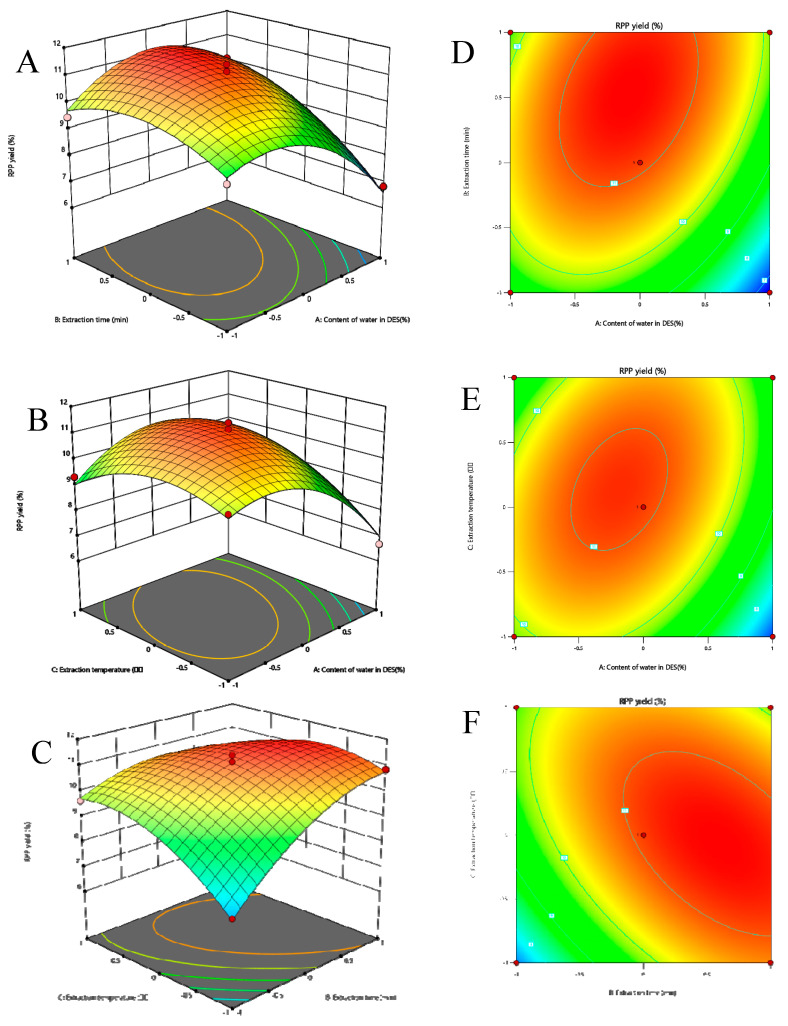
Three−dimensional response surface plots of deep eutectic solvent assisted extraction (**A**–**C**); two-dimensional contour plots of deep eutectic solvent-assisted extraction (**D**–**F**).

The response surface and higher line plots were plotted using the Design-Expert 13 software. Fixing one of the three variables as the median value, the effect surface plots of the extraction rate were plotted. From Figure 2A–C, it can be seen that the B effect surface was steeper compared with the C direction, indicating that the extraction time had a greater effect on the extraction rate than the water content and extraction temperature. The interaction between these factors was significant based on the shapes of the contour lines and the steepness of the response curves shown in Figure 2D–F.

### 2.2. Scanning Electron Microscopy (SEM) Image

**Figure 3:**
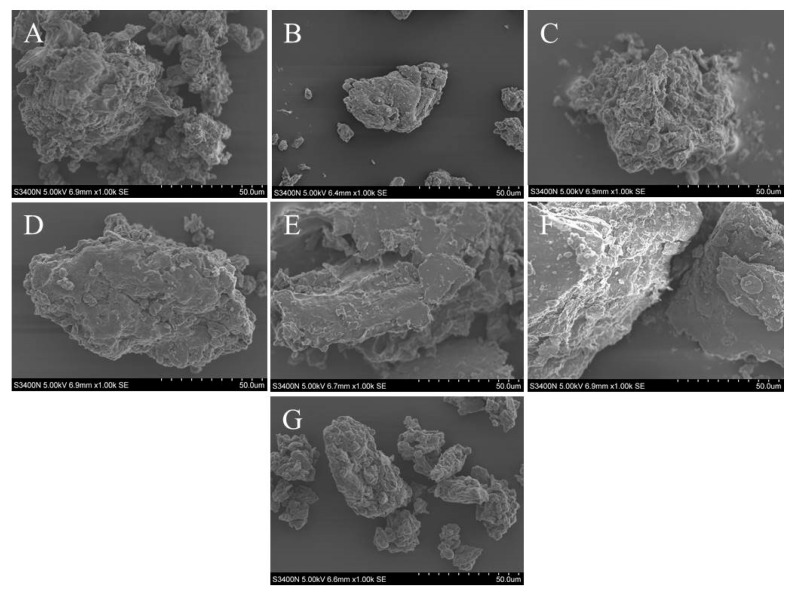
The powder after different methods extraction exhibited various degrees of fragmentation with 1.00k SEM: (**A**) ultrasound-assisted DES-6 extraction; (**B**) decoction piece; (**C**) ultrasonic water extraction; (**D**) distilled water extraction; (**E**) hot water reflux; (**F**) thermal extraction; (**G**) the untreated raw medicinal powder.

The cellular destruction of the sample powders is shown in Figure 3. Ultrasound-assisted extraction with DES-6 disrupted the cells to a greater extent than aqueous extraction, leading to the improved extraction of compounds from the dried powder. Consequently, the combination of DES-6 and ultrasonication afforded superior efficacy as the optimal method for polysaccharides extraction from Radix *Peucedani*.

**Figure 4:**
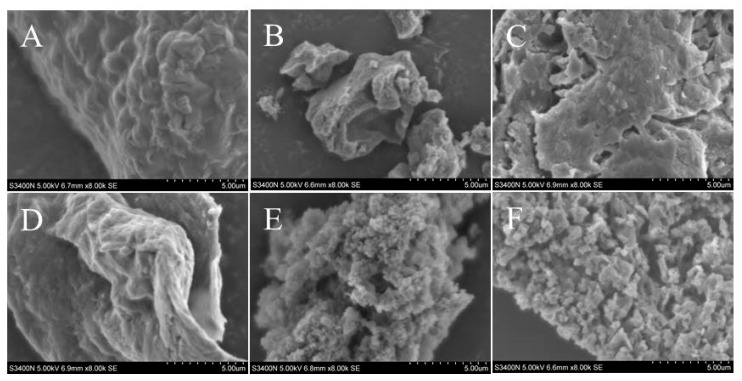
The polysaccharides morphology using different extraction methods with 8.00k SEM. (**A**) ultrasound-assisted DES-6 extraction; (**B**) decoction piece; (**C**) ultrasonic water extraction; (**D**) distilled water extraction; (**E**) hot water reflux; (**F**) thermal extraction.

The morphologies of the polysaccharides extracted from Radix *Peucedani* by different methods were observed using SEM. As shown in Figure 4, the different polysaccharide extraction methods led to various morphological differences [25]. The polysaccharides’ morphology was fragmented, granular, flocculent and verrucose, and the whole showed complete dispersion, and fold-like structures could be seen after magnification. It has been reported that the microstructure of polysaccharides is a pore-like or network structure with good rheological properties [27]. (E) and (F) were more complete as a whole, but cracks were present and the surface was uneven. (C) contained a small amount of fragmentation, with poor surface smoothness and the presence of bumps and depressions. (D) and (B) were agglomerated forms by a large number of blocky structures that were coiled and zigzagged, with the presence of a clear coarse band structure at the edge of the structure observed after 8000× magnification, which suggests that there were a large number of molecules with a lax internal structure. (A) displayed an irregular tumor-like structure, with one “tumor” of different sizes and round or oval shapes clustered together, with well-defined edges and smooth surfaces.

### 2.3. Fourier-Transform Infrared (FT-IR) Spectroscopy

**Figure 5:**
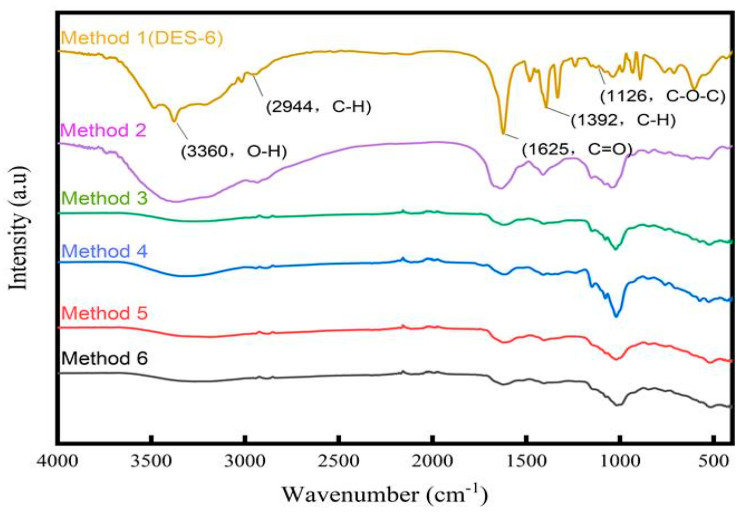
FT−IR spectra of Radix *Peucedani* polysaccharides from different extraction methods.

The mid-infrared absorption of molecules involves a jump between the vibrational energy states and the rotational substates of the molecule, which can used to elucidate the molecular structure [28]. As shown in Figure 5, the polysaccharides displayed two intense absorption peaks in the 1100–1010 cm^−1^ range. It is presumed that one of the furanose rings was present [29]. Plant polysaccharides are often composed of long chains containing multiple α- or β-glycosidic bonds between monosaccharide units of the same or different types [30]. The signal near 891 cm^−1^ corresponded to the vibration of β-glycosidic bonds, whereas the absence of an absorption peak near 847 cm^−1^ indicated a lack of α-glycosidic bonds. In addition, FT-IR spectroscopy can be used to evaluate the functional groups present in polysaccharides [31]. For example, a broad peak at 3360 cm^−1^ corresponds to O–H bond stretching, while strong absorption at 1625 cm^−1^ is indicative of C=O stretching. In addition, peaks at 1392 and 2944 cm^−1^ correspond to C–H bending and C–H stretching, respectively [32]. A band in the region of 1160–1070 cm^−1^ is indicative of C–O–C bonds, and absorption peaks in the region of 930–800 cm^−1^ are associated with a number of linked polysaccharide features such as the D-barellan glucose ring and a-type glycan bonds [33]. As shown in Figure 5, the IR spectrum of the polysaccharides extracted with DES-6 exhibited a similar pattern to that obtained using Method 2–6. The majorly peaks were almost identical, indicating that the extraction process using DES-6 did not induce a shift in the main functional groups [34].

### 2.4. Antioxidant Activity

#### 2.4.1. Scavenging of 2,2-Diphenyl-1-picrylhydrazyl (DPPH) Radicals

**Figure 6:**
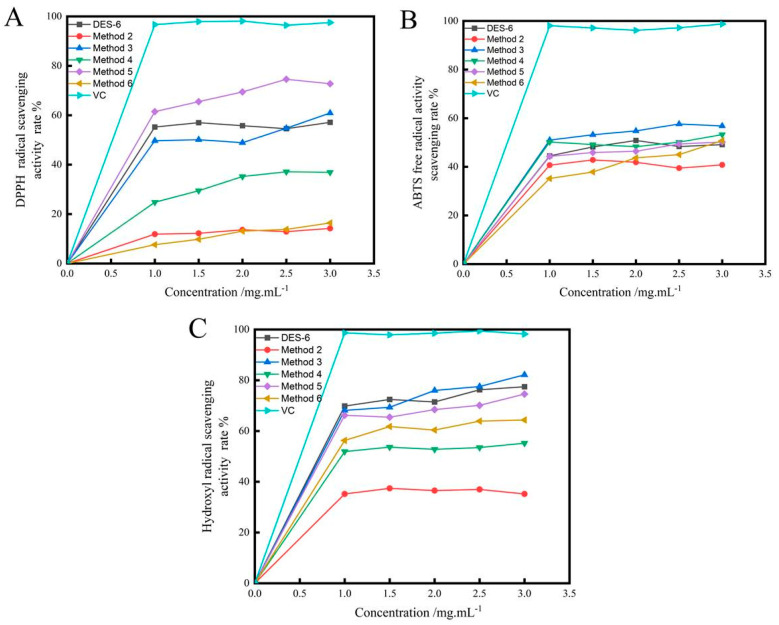
Antioxidant activity of RPP on scavenging activity to DPPH radicals (**A**); scavenging activity to ABTS radicals (**B**) and scavenging activity to hydroxyl radicals (**C**).

DPPH is often employed as a representative reagent to examine the free radical scavenging activities of bioactive compounds. The antioxidant activity of polysaccharides is attributed to their hydrogen-donating capacity [35]. The antioxidant activities of the polysaccharides extracted from Radix *Peucedani* using different methods are shown in Figure 6A. The results demonstrated that the DPPH radical scavenging activities of the Radix *Peucedani* polysaccharides were dose dependent across the evaluated concentration range (1–3 mg·mL^−1^). The DPPH radical scavenging activity of the extract obtained using Method 5 increased from 61.45% to 72.76%. This was almost three times that observed for the extracts obtained using Method 2 (14.2%) and Method 6 (16.4%).

#### 2.4.2. Scavenging of 2,2′-Azino-bis(3-ethylbenzothiazoline-6-sulfonic acid) (ABTS)radicals

The ABTS assay is another versatile tool for measuring the in vitro antioxidant activities of compounds. As shown in Figure 6B, the obtained results were similar to those observed for the scavenging of DPPH radicals. The ABTS radical scavenging activity of the samples increased at higher concentrations.

#### 2.4.3. Scavenging of Hydroxyl Radicals

Hydroxyl radicals are an important reactive oxygen species that can cause cell death in the body. The hydroxyl radical scavenging results observed for the Radix *Peucedani* polysaccharides are shown in Figure 6C. The scavenging of hydroxyl radicals was dose dependent, with scavenging rates of 77.455%, 35.21%, 82.15%, 55.21%, 74.56% and 64.35%, respectively, at a dose of 3 mg·mL^−1^. These results demonstrate that the polysaccharides exhibited strong hydroxyl scavenging ability.

### 2.5. Chemical Modification

#### 2.5.1. Molecular Weight

Molecular weight is an easily adjustable structural feature of polysaccharides that affects their utilization and biological activity. Polysaccharides with low molecular weights have weaker intramolecular hydrogen bonding and more free amino and hydroxy groups, which are favorable for their biological activity [36]. As shown in Table S3 (Supplementary Materials), the width index of the molecular weight distribution for the Radix *Peucedani* polysaccharides was measured to be 3.591 and the weight-average molecular weight was 3.582 × 10^3^ kDa. One point to be noted is that natural polysaccharides can undergo physicochemical and functional changes during the preparation process, and polysaccharides prepared using different methods often display different monosaccharide compositions and average molecular weights [37]. Thus, it is necessary to apply suitable methods to efficiently obtain bioactive polysaccharides of a certain molecular weight range.

**Figure 7:**
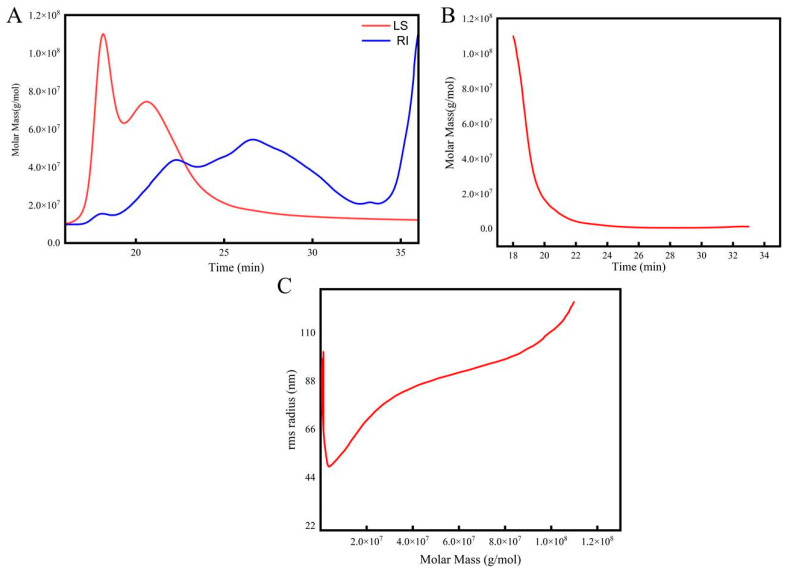
The light scattering signal (**A**); absolute molecular weight analysis of RPP (**B**); molecular conformation analysis of RPP (**C**).

As shown in Figure 7A,B, the absolute molecular weight analysis of the Radix *Peucedani* polysaccharides by multiangle laser light scattering revealed two symmetrical peaks (red line, i.e., LS, in V), along with four peaks for the difference signal (blue line, i.e., RI, in RIU). The molar mass was mainly distributed between 1.0 × 10^6^ and 1.0 × 10^8^ g·mol^−1^. For the same polysaccharides, the narrower the molar mass distribution, the less it deviates from a Newtonian fluid and the weaker the pseudoplasticity [38].

#### 2.5.2. Polysaccharide Molecular Conformation

The molecular conformation of the Radix *Peucedani* polysaccharides was evaluated by using size-exclusion chromatography with multiangle laser light scattering index detection to assess the relationship between the molar mass and molecular radius. The analyzed molecular conformation is shown in Figure 7C, where the molecular conformation diagram was plotted using the molar mass (g·mol^−1^) as the horizontal coordinate and the root-mean-square radius (R.M.S. radius, nm) as the vertical coordinate. The slope of the molecular conformation plot was 0.03 + 0.00, and the plot displayed a U-shaped curve, in accordance with previous results for highly branched polysaccharides [39].

### 2.6. Monosaccharaide Composition

**Figure 8:**
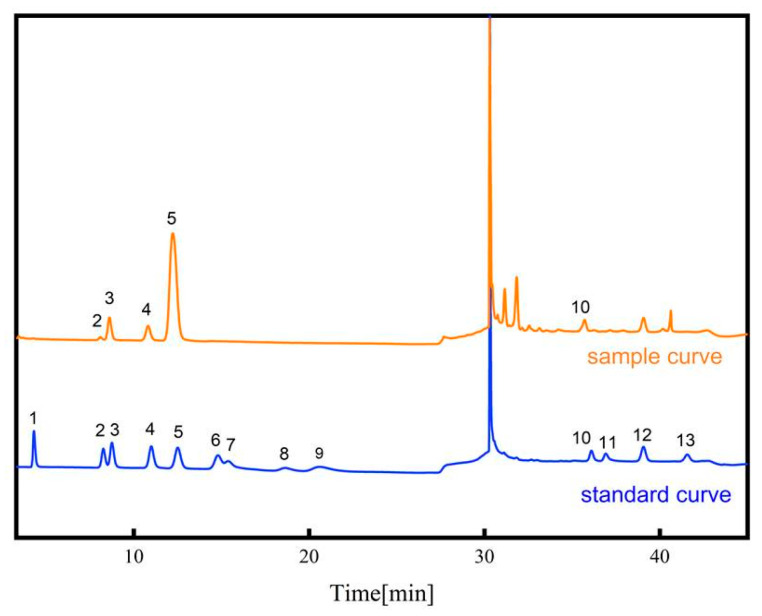
Chromatogram of mixed standard curve and the sample curve: 1—Fucose; 2—Rhamnose; 3—Arabinose; 4—Galactose; 5—Glucose; 6—Xylose; 7—Mannose; 8—Fructose; 9—Ribose; 10—Galacturonic acid; 11—Guluronic acid; 12—Glucuronic acid; 13—Mannuronic acid.

The standard curve method was employed to calculate the contents of 13 monosaccharides in the samples and the relative amount of each monosaccharide with respect to the total monosaccharide content, and the results are presented in Table S4 (Supplementary Materials). As shown in Figure 8, the RPP contained five monosaccharides, namely, rhamnose, galacturonic acid, glucose, galactose and arabinose. But there were some variations in the contents and proportional compositions of the five monosaccharides. The most abundant monosaccharide was glucose, which accounted for more than 60% of the total followed by galacturonic acid, whereas rhamnose was the least abundant.

To the best of our knowledge, this study represents the first time that Radix *Peucedani* polysaccharides have been comprehensively characterized using three analytical techniques: FT-IR spectroscopy, SEM and ICS. FT-IR spectroscopy was employed to obtain the infrared absorption spectra of the polysaccharides, enabling identification of their molecular structure and chemical groups. SEM was utilized to characterize the morphologies of the polysaccharides and obtain information regarding their shapes, surface topographies and other characteristics. Finally, ICS was used to determine the compositions of the polysaccharides and relative abundances of the constituent monosaccharides with high precision. The combined application of these techniques provided crucial information regarding the structures, morphological features and compositions of the polysaccharides, thus establishing a reliable dataset for their future development and applications. The obtained findings are essential for investigating the nutritional composition, pharmacological activities and quality control of Radix *Peucedani* polysaccharides.

### 2.7. Pharmacological Effects of Polysaccharides Antioxidant Networks

#### 2.7.1. Venny Diagram Construction

The Venny platform (https://www.bioinformatics.com.cn/static/others/jvenn/example.html accessed on 14 July 2023) was used to obtain the intersection of ingredient target and disease targets, and the common targets of the two were screened as potential targets for the antioxidant activities of thepolysaccharides fractions. The obtained Venny diagram is presented in Figure S1 (Supplementary Materials).

#### 2.7.2. Target Screening and Enrichment Analysis

A protein–protein interaction (PPI) network was constructed, comprising 185 edges and 41 nodes, as shown in Figure S2 (Supplementary Materials). In this network, the nodes represent potential targets of action, which were ranked based on their node degree. Among them, interleukin-1 beta (IL1B), caspase-3 (CASP3) and peroxisome proliferator-activated receptor gamma (PPARG) were the top three targets, indicating that these proteins may play important roles.

The results were visualized by Gene Ontology (GO) functional enrichment analysis, which revealed 223 biofunctional entries, among which, most of the entries were related to biological processes (BP) (biological processes), especially the positive regulation of apoptotic process term. There were 22 entries related to cell components (CC) and 43 entries related to molecular functions (MF). According to the *p* values and number of enriched genes, the top entries of BP-, CC- and MF-enriched genes were selected and plotted as bar graphs, and the results are presented in Figure S3 (Supplementary Materials). KEGG pathway enrichment analysis revealed that the Radix *Peucedani* polysaccharides affected a total of 56 antioxidant signaling pathways. The above pathways were screened on the basis of *p* values and high numbers of enriched genes, and the top 20 pathways were selected and plotted as a bubble map, as shown in Figure S4 (Supplementary Materials).

#### 2.7.3. Constructing the “Herb-Polysaccharide Fractions-Target-Pathway” Network

**Figure 9:**
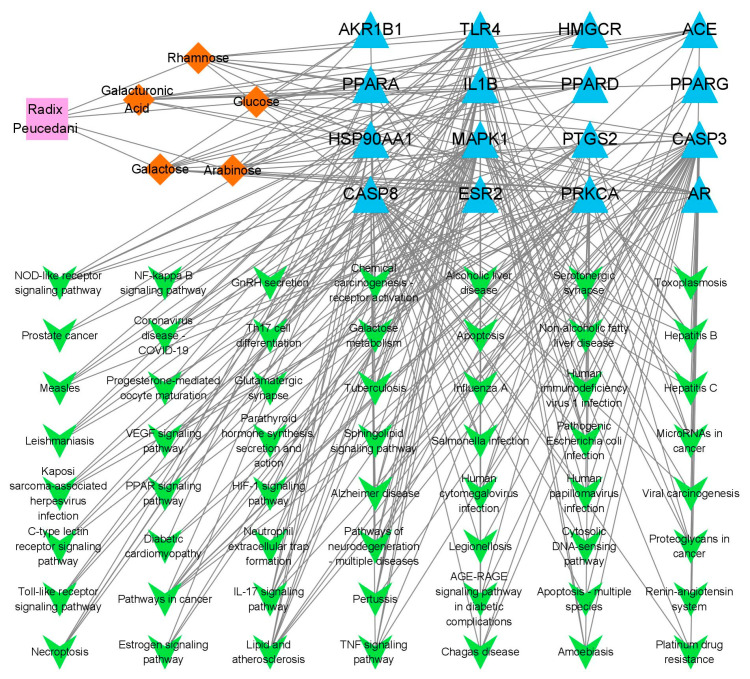
“Herb-Active Ingredient-Target-Pathway” network diagram.

As shown in Figure 9, purple nodes represented herbs, orange nodes represented polysaccharide components with a total of five, blue nodes represented the 16 core targets and green nodes represented pathways with a total of 56. Network Analyzer was performed based on the degree values of polysaccharide components, potential targets and the average of BC and CC, which finally yielded the core targets. MAPK1 (mitogen- activated protein kinase 1), CASP3 (caspase-3) and IL1B (beta interleukin 1, beta) and two key polysaccharide fractions, namely, glurose and galactose. Analysis of the network diagram revealed that multiple targets could be influenced by the same component and, conversely, that a single target could be affected by multiple components. This suggests that the antioxidant effects of Radix *Peucedani* polysaccharides may be mediated by a diverse range of components targeting multiple pathways. Previous research has indicated that the proinflammatory cytokine IL1B plays a role in antioxidant activity based on the expression patterns of HaCaT cells [40] and that activation of the MAPK pathway is the central event in UV-induced intracellular signaling [41]. These results show that the results of network pharmacology prediction are closer to the existing related literature, indicating that this methodology has a certain degree of accuracy and reliability and, at the same time, it is also compatible with the holistic nature of traditional Chinese medicine. However, there remain some limitations in network pharmacology, and the results still need to be validated by specific pharmacokinetic, pharmacodynamic and safety studies at both the animal and cellular levels.

#### 2.7.4. Component-Target Molecular Docking Analysis

**Figure 10:**
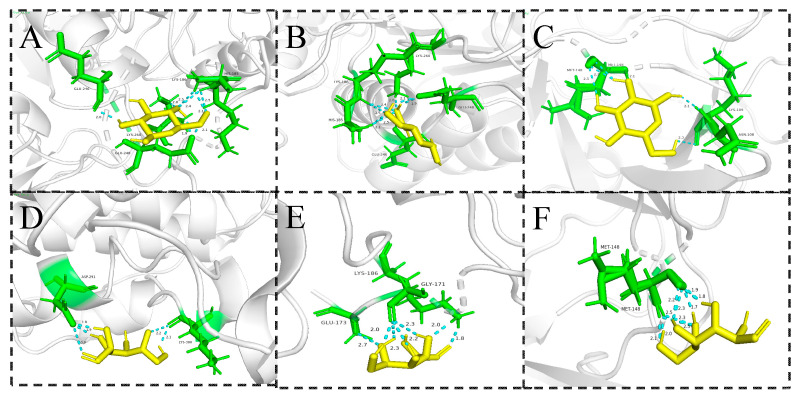
Molecular docking diagram. The docking result of (**A**) glucose and MAPK1; (**B**) glucose and CASP3; (**C**) glucose and IL1B; (**D**) galactose and MAPK1; (**E**) galactose and CASP3; (**F**) galactose and IL1B.

The top three core targets with the highest degree values screened in Section 2.7.3 were MAPK1, CASP3 and IL1B, and the two polysaccharide components with the highest degree values were sequentially verified by molecular docking using AutoDock Tools 1.5.7 software, and the results are shown in Table S5 (Supplementary Materials). The binding activities of glucose and IL1B, and that of galactose and CASP3, were higher than others, indicating that the action of the above components on these targets may be a potential mechanism for the antioxidant effects exerted by Radix *Peucedani*, which were visualized and analyzed using PyMOL 2.5 software (Figure 10).

The visualization results showed that the polysaccharides fractions primarily established hydrogen bonds at the binding sites of five hydrophilic amino acid residues, LYS (lysine), GLY (glycine), ASN (asparagine), ASP (aspartic acid) and HIS (histidine), and formed hydrophobic interactions with two hydrophobic amino acids, MET (methionine) and GLU (glycine).

## 3. Materials and Methods

### 3.1. Plant Materials and Chemicals

Radix *Peucedani* decoction pieces were provided by Hongya County Waya Mountain Pharmaceutical Co., Ltd. (Origin: Zhejiang, China, Batch No.: 220801, Production Date: 4 August 2022, Drug Manufacturing License No.: Chuan 20160063; Execution Standard: Pharmacopoeia of the People’s Republic of China 2020 Edition). The reagents used in the experiment are listed in Table S6 (Supplementary Materials). All the above reagents are pure analytical reagents. Water is distilled water.

### 3.2. Experimental Methods

#### 3.2.1. Preparation of DESs

The nine DES systems shown in Table S7 (Supplementary Materials). The process of preparing DES is described in Section S1.1 (Supplementary Materials).

#### 3.2.2. Powder Pretreatment

Radix *Peucedani* decoction pieces were ground into samples and defatted by refluxing with petroleum ether (boiling range of 30–60 °C) at a ratio of 1:4 (*w*/*v*) for 1 h using reflow reactor equipment. The resulting mixture was then subjected to vacuum concentration. Alcohol precipitation was carried out by adding 4–5 times the volume of 80% ethanol (repeated twice), followed by centrifugation at 12,000 rpm for 10 min using a centrifuge. The filter residue was dried using hot air.

#### 3.2.3. Extraction of Radix Peucedani Polysaccharides

Deproteinization of polysaccharides from Radix *Peucedani* was performed using the trichloroacetic acid-n-butanol method [34]. The resulting supernatant was collected and stored at 4 °C for further testing. Details of the six different methods used for extracting Radix *Peucedani* polysaccharides can be found in Section S1.2 of the Supplementary Materials.

#### 3.2.4. Plotting of Standard Curves for Polysaccharides Content

The content of polysaccharides was determined using the phenol-sulfuric acid method [42,43]. The specific methodology and formulas are described in Section S1.3 (Supplementary Materials).

### 3.3. Process Optimization for Polysaccharides Extraction by Ultrasound-Assisted DESs

#### 3.3.1. Single-Factor Test

The extraction rates of different types of DES were compared and five extraction parameters were optimized for Radix *Peucedani* polysaccharides (see Section S1.4 in the Supplementary Materials).

#### 3.3.2. Response Surface Optimization Test

Based on previous studies [44], the results of the single-factor experiments on polysaccharides content indicated that three factors strongly influence the polysaccharide content. The range of experimental variables for these factors is shown in Table S8 (Supplementary Materials). The response surface optimization test consisted of 17 independent experiments, with each treatment being performed in triplicate.

### 3.4. Structural Analysis of Polysaccharides

#### 3.4.1. Scanning Electron Microscopy (SEM) Analysis

The cellular destruction morphology of the Radix *Peucedani* powders extracted by different methods and obtained polysaccharides were observed using a Scanning Electron Microscope (SEM) [45].

#### 3.4.2. Fourier Transform Infrared (FT-IR) Spectra

Prehistoric polysaccharides were milled and pressed into granular form in order to perform FT-IR measurements. The frequency range used was 400–4000 cm^−1^ to detect functional groups.

#### 3.4.3. Evaluation of Antioxidant Activity

The DPPH radical scavenging activity was conducted according to previous reports [46] with slight modifications. The hydroxyl radical scavenging activity was performed using previous methods [47]. Reference to previous studies [48] was made to determine the ABTS radical cation scavenging activity.

#### 3.4.4. Monosaccharide Analysis

The polysaccharides were hydrolyzed following the methods described in the literature [49]. The configuration of the assay instrument and the mixed control is mentioned in the Section S1.5 (Supplementary Materials). The absolute molecular weight of the samples was measured using gel chromatography, laser light scattering and an oscillometric detector in tandem [50]. Further details can be found in Section S1.6 of the Supplementary Materials.

### 3.5. Network Pharmacology Analysis

#### 3.5.1. Prediction and Intersection of Targets

The polysaccharides fraction proteins were corrected to standard gene names through the UniProt database (https://www.uniprot.org/ accessed on 18 July 2023) [51]. In total, 166 target genes and 1132 disease genes were obtained from the database. The specific databases used are mentioned in detail in Section S1.7 (Supplementary Materials).

#### 3.5.2. Network Creation and Enrichment Analysis

The STRING (https://string-db.org/ accessed on 24 July 2023) database was used to analyze the protein–protein interaction (PPI) networks and download the data. The data was then imported into Cytoscape 3.9.1 software (Boston, MA, USA) to construct the PPI network. After then, Gene Ontology (GO) and Kyoto Encyclopedia of Genes and Genomes (KEGG) pathway enrichment of the antioxidant targets of Radix *Peucedani* using the David database (https://david.ncifcrf.gov/ accessed on 26 July 2023) were analyzed [52]. Finally, the “Herb-Polysaccharide component-Target-Pathway” network was constructed using Cytoscape 3.9.1 software.

#### 3.5.3. Molecular Docking

The target structures were combined with 3D protein structure data from the Protein Data Bank (PDB, https://www.rcsb.org accessed on 1 August 2023) using AutoDock Tools 1.5.7 software for ligand docking. Finally, PyMOL 2.5 (New York, NY, USA) was used to visualize the docking results.

### 3.6. Statistical Analysis

Box–Behnken test and analysis of variance were performed on Design-Expert 13. Origin 2021 was used to graph the experimental data and chromatographic data.

## 4. Conclusions

In our study, an efficient, simple and environmentally friendly ultrasound-assisted extraction method based on DES was applied to extract Radix *Peucedani* polysaccharides. DES-6 consisting of betaine with 1, 2-propanediol (molar ratio of 1:2) was considered the best extraction solvent with a higher extraction rate. The molecular weight of the RPP was measured to be about 3.582 × 10^3^ kDa, consisting of five monosaccharides, namely, rhamnose, galacturonic acid, glucose, galactose and arabinose, with the molecular conformation showing a “U” shape curve. Furthermore, the polysaccharides extracted from Radix *Peucedani* using ultrasound-assisted DES-6 showed favorably antioxidant activity. The results of network pharmacology as well as molecular docking showed that Radix *Peucedani* polysaccharides could exert antioxidant effects through the core components such as glucose and galactose, which could act on the core targets such as MAPK1, CASP3 and IL1B.

## Figures and Tables

**Table 1:** Variance Analysis of the Regression Model.

Source	Sum of Squares	df	Mean Square	F Value	*p*-Value	Significant
Model	34.45	9	3.83	43.31	<0.0001	significant
A	3.25	1	3.25	36.81	0.0005	**
B	6.6	1	6.6	74.67	<0.0001	**
C	1.2	1	1.2	13.62	0.0078	*
AB	2.14	1	2.14	24.19	0.0017	*
AC	2.38	1	2.38	26.97	0.0013	*
BC	2.78	1	2.78	31.43	0.0008	**
A^2^	8.53	1	8.53	96.54	<0.0001	**
B^2^	2.65	1	2.65	30.01	0.0009	**
C^2^	3.36	1	3.36	38.02	0.0005	**
Residual	0.6187	7	0.0884			
Lack of Fit	0.4426	3	0.1475	3.35	0.1365	not significant
Pure Error	0.176	4	0.044			
Cor Total	35.07	16				

Note: *, significant difference, *p* < 0.05; **, highly significant difference, *p* < 0.001.

## Data Availability

The data presented in this study are available on request from the corresponding author.

## Supplementary Materials

The following supporting information can be downloaded at: https://www.mdpi.com/article/10.3390/molecules28237845/s1, Figure S1: Venn diagram of drug targets and antioxidant targets; Figure S2: Visual PPI network diagram of antioxidant for Radix *Peucedani* polysaccharides; Figure S3: Analysis diagram of GO functional enrichment; Figure S4: Enrichment analytic diagram of KEGG pathway; Table S1: Results of single-factor experiments; Table S2: The Box-Behnken design with independent variables and observed values of the extraction yield of polysaccharides from Radix *Peucedani*; Table S3: Molecular structural characteristic parameters of RPP; Table S4: Monosaccharide composition and content; Table S5: Binding energies of the key polysaccharides fraction to the core targets; Table S6: All reagents used in the experiment; Table S7: Composition of deep eutectic solvents (DESs); Table S8: Response surface test factors and levels; Table S9: Elution gradients for anion-exchange chromatography; Section S1.1: Preparation of DESs; Section S1.2: Extraction of Radix *Peucedani* polysaccharides; Section S1.3: Determination of polysaccharides content; Section S1.4: Single-factor test; Section S1.5: Carbohydrate composition; Section S1.6: Molecular Weight and molecular conformation of monosaccharides; Section S1.7: Prediction and intersection of targets.
