# Supplementary material for: Polysaccharides from Radix Peucedani: Extraction, Structural Characterization and Antioxidant Activity
Source: Molecules. 2023 Nov 29;28(23):7845. doi: 10.3390/molecules28237845 (PMC10707930; doi:10.3390/molecules28237845)
Supplement: Supplementary file 1 [file molecules-28-07845-s001.zip › molecules-2741933-supplementary.pdf]

molecules

MDPI

Supplementary Materials

Polysaccharides from Radix *Peucedani*: Extraction, Structural Characterization and Antioxidant Activity

Jie Zhang, Chenyue Wang, Qian Li * and Wei Liang

State Key Laboratory of Aridland Crop Science, College of Agronomy, Gansu Agricultural University, Lanzhou 730070, China; jie.zhang101@icloud.com (J.Z.); wcy04042023@163.com (C.W.); liangw@gsau.edu.cn (W.L.)
* Correspondence: liqian1984@gsau.edu.cn

Citation: Zhang, J.; Wang, C.; Li, Q.; Liang, W. Polysaccharides from Radix *Peucedani*: Extraction, Structural Characterization and Antioxidant Activity. *Molecules* **2023**, *28*, 7845. https://doi.org/10.3390/molecules28237845

Academic Editor: Paola Di Donato

Received: 12 November 2023
Accepted: 27 November 2023
Published: 29 November 2023

Copyright: © 2023 by the authors. Licensee MDPI, Basel, Switzerland. This article is an open access article distributed under the terms and conditions of the Creative Commons Attribution (CC BY) license (https://creativecommons.org/licenses/by/4.0/).

S1. Materials and methods

S1.1. Preparation of DESs

The hydrogen bond acceptor (choline chloride and anhydrous betaine) and hydrogen bond donor (xylitol, 1, 2-propanediol glycol, urea, lactic acid, citric acid) were mixed according to table 1 and placed in a conical flask with a magnetic stirrer. The reactions were maintained at a temperature of 80 ℃ and stirred for 40 min until the solution entered a transparent state. It was left sealed at room temperature for one week; the liquid was still homogeneous and transparent.

S1.2. Extraction of Radix Peucedani polysaccharides

Method 1 (ultrasound-assisted DESs): 1 g of the pretreated Radix *Peucedani* powder was weighed and added to 20 mL of different DES solvents. The sonication time was 30 min, power 240W, temperature 45 ℃ and water content 30%. After centrifugation at 12,000 rpm for 10 min, the supernatant was collected in a 25 mL volumetric flask. Radix *Peucedani* polysaccharides powder were obtained (RPP).

Method 2 (decoction piece): 1 g of Radix *Peucedani* decoction pieces was weighed and added to 20 mL of distilled water. It was extracted using a magnetic stirrer 800 rpm in boiling water for 30 min, and the extraction was repeated twice. The extracts were combined and treated by deproteinization and left to be measured.

Method 3 (ultrasonic water extraction): 1 g of the pretreated Radix *Peucedani* powder was weighed and added to 20 mL of distilled water. The sonication time was 30 min, power 240 W, temperature 45 ℃. The supernatant was deproteinized and left to be measured.

Method 4 (distilled water extraction): 1 g of the pretreated Radix *Peucedani* powder was weighed and added to 20 mL of distilled water. Using a magnetic stirrer 800 rpm, cold water extraction for 30 min. The supernatant was deproteinized and left to be measured.

Method 5 (hot water reflux): 1 g of the pretreated Radix *Peucedani* powder was weighed and added to 20 mL of distilled water and extracted by hot reflux at 45 ℃ for 30 min. The supernatant was deproteinized and left to be measured.

Method 6 (thermal extraction): 1 g of the pretreated Radix *Peucedani* powder was weighed and added to 20 mL of distilled water. Use magnetic stirrer 800 rpm, it was heated to 45 ℃ and hot water extracted for 30 min. The supernatant was deproteinized and left to be measured.

S1.3. Determination of polysaccharides content

The glucose standard curve was plotted with the polysaccharides concentration as the horizontal coordinate (x) and the measured absorbance as the vertical coordinate (y).

Hepreparation of standard curve for glucose standard curve was obtained as regression equation: y=0.36194x-0.00176, R^2=0.9991.

Determination of polysaccharides content in RPP: the polysaccharides content was calculated as described in Equation (1).

The yield of RPP (%)=$\frac{(C \cdot V)}{M} \cdot 100\%$ (1)

In the formula: C is the polysaccharides concentration in the extract; V is the volume of the diluted extract; and M is the mass of the extracted sample.

S1.4. Single-factor test

Precisely quantified 1 g of the pretreated Radix *Peucedani* powder and different species of DES were added to the ultrasonic extraction vessel. In order to screen the extraction rate of polysaccharides from Radix *Peucedani*, the extraction rates of polysaccharides from different types of DES were compared. The extraction parameters, including content of water in DES (10, 20, 30, 40 and 50%), extraction time (10, 20, 30, 40 and 50 min), extraction temperature (25, 35, 45, 55 and 65 °C), ultrasonic power (240, 300, 360, 420 and 480W) and solid–liquid ratio (10, 20, 30, 40 and 50 ml) were optimized to maximize the extraction efficiency.

S1.5. Carbohydrate composition

The chromatographic system used a Thermo ICS 5000+ ion chromatography system (ICS 5000+, Thermo Fisher Scientific, USA), and the monosaccharide fractions were analyzed and detected using an electrochemical detector. A Dionex™ CarboPac™ PA20 (150*3.0 mm, 10 μm) liquid chromatography column was used, and the injection volume was 5 μL. The mobile phase A (H_2O), mobile phase B (0.1 mol.L^{-1} NaOH), mobile phase C (0.1 mol.L^{-1} NaOH, 0.2 mol.L^{-1} NaAc) were used at a flow rate of 0.5 ml. min^{-1}, the column temperature was 30 °C, and the elution gradient was as shown in Table S9 (supporting information).

The 13 kinds of monosaccharide standards were weighed accurately in a 10 mL volumetric flask and added to water to form a 10 mg. mL^{-1} standard solution; the appropriate amount of the standardly solution was mixed to form a suitable concentration of the mixing standard.

S1.6. Molecular Weight and molecular conformation of monosaccharides

The sample was dissolved in 0.1 mol.L^{-1} $NaNO_3$ aqueous solution (containing 0.02% NaN_3, w/v) at a final concentration of 1 mg.mL^{-1} and filtered through a filter with a pore size of 0.45 μm for on-line detection. A gel exclusion chromatographic column, Ohpak SB-805 HQ (300×8 mm) and Ohpak SB-803 HQ (300×8 mm), was used in series. The column temperature was 45 °C, injection volume was 100 μL, mobile phase (0.02% NaN_3, 0.1 mol.L^{-1} $NaNO_3$), flow rate was 0.6 mL. min^{-1} and the elution gradient: isocratic 75 min.

S1.7. Prediction and intersection of targets

The five polysaccharides fractions contained in Radix Peucedani were searched in the Traditional Chinese Medicine Systems Pharmacology Database and Analysis Platform database (TCMSP, https://old.tcmsp-e.com/tcmsp.php) and Swiss Target Prediction database (http://www.swisstargetpr ediction.ch/), and the polysaccharides fraction proteins were corrected to standard gene names through the UniProt database (https://www.uniprot.org/). A total of 166 target genes were summarized by de-emphasis. The human gene databases GeneCards (http://www.genecards.org/), Online Mendelian Inheritance in Man database (OMIM, https://www.omim.org/) and PharmGkb (https://www.pharmgkb.org/) were searched for target genes related to keyword "Antioxidant". The results of the three databases were combined and de-emphasized; 1,132 disease targets were finally obtained.

Table S1. Results of single-factor experiments.

Single-factor experiments						
Factor 1	DESs Water content (%)	10	20	30	40	50
	Yield (%)	8.53	10.53	9.7	9.43	8.53
Factor 2	Withdrawal times (min)	10	20	30	40	50
	Yield (%)	7.53	8.43	10.43	9.43	8.7
Factor 3	Ultrasonic power (W)	240	300	360	420	480
	Yield (%)	8.6	9.6	10.46	10.73	10.46
Factor 4	Extraction temperatures (℃)	25	35	45	55	65
	Yield (%)	7.43	8.93	9.7	10.3	9.5
Factor 5	Material–liquid ratios (%)	10	20	30	40	50
	Yield (%)	8.43	8.5	10.6	9.6	9.5

Table S2. The Box–Behnken design with independent variables and observed values of the extraction yield of polysaccharides from Radix *Peucedani*.

No.	Factor			RPP yield /%
	A（%）	B（min）	C（℃）	
1	-1	1	0	9.4526
2	1	-1	0	6.82968
3	0	0	0	10.8252
4	0	0	0	11.1382
5	1	0	-1	6.67652
6	-1	-1	0	9.1228
7	1	0	1	9.1487
8	-1	0	1	9.3245
9	0	0	0	11.3843
1	0	1	1	9.8011
11	0	0	0	11.1325
12	0	-1	-1	7.3368
13	0	-1	1	9.6268
14	-1	0	-1	9.9403
15	1	1	0	10.0836
16	0	0	0	10.9668
17	0	1	-1	10.8446

Table S3. Molecular structural characteristic parameters of RPP.

Parameters		RPP
Molecular weight (g/mol)	Mw	3.528x103
	Mn	9.82x102
	Mz	3.2960x104
Polydispersity	MW/Mn	3.591
	Mz/Mn	33.546
RMS radius moments (nm)	Rw	76.9
	Rn	83.2
	Rz	80.1

Table S4. Monosaccharide composition and content.

Monosaccharide name	Test results(ug/mg)	Percentage(%)	Mole–mass ratio(%)
Fuc	0	0.00%	0.00%

Rha	0.6596	1.54%	1.68%
Ara	3.8281	8.94%	10.64%
Gal	2.7349	6.38%	6.33%
Glc	28.8934	67.45%	66.91%
Xyl	0	0.00%	0.00%
Man	0	0.00%	0.00%
Fru	0	0.00%	0.00%
Rib	0	0.00%	0.00%
Gal-UA	6.7235	15.69%	14.45%
Gul-UA	0	0.00%	0.00%
Glc-UA	0	0.00%	0.00%
Man-UA	0	0.00%	0.00%

Table S5. Binding energies of the key polysaccharides fraction to the core targets.

Chemical compound	MAPK1 /$kJ\cdot mol^{-1}$	CASP3 /$kJ\cdot mol^{-1}$	IL1B /$kJ\cdot mol^{-1}$
Glucose	-3.19	-3.51	-3.74
Galactose	-0.8	-1.79	-1.74

Table S6. All reagents used in the experiment.

Manufacturer	Reagent Name
Shanghai McLean Biochemical Science and Technology Co., Ltd. (Shanghai, China)	Choline chloride, betaine anhydrous, xylitol, 1,2-propylene glycol, urea, and lactic acid
Sinopharm Chemical Reagent Co., Ltd. (Shanghai, China)	D-glucose, phenol, sulfuric acid, petroleum ether, trichloroacetic acid, n-butanol, hydrated citric acid, sodium nitrate, 1,1-diphenyl-2-trinitrophenylhydrazine, ethanol, ferrous sulfate, salicylic acid, 30 % hydrogen peroxide, diammonium 2,2'-azino-bis (3-ethylbenzothiazoline-6-sulfonate) (ABTS), potassium persulfate and l (+) - ascorbic acid (VC)
Sigma-Aldrich (Shanghai, China)	Fucose, rhamnose, arabinose, galactose, glucose, xylose, mannose, fructose, ribose, galacturonic acid, glucuronic acid, mannuronic acid, glucuronic acid, sodium hydroxide, sodium acetate standard, dimethyl sulfoxide
ANPEL (Shanghai, China)	Trifluoroacetic acid and methanol

Table S7. Composition of deep eutectic solvents (DESs).

No	HYdrogen bond acceptor	Hydrogen bond donor	Molar ratio
1	Choline chloride	1,2-Propylene glycol	1:2
2	Choline chloride	Urea	1：2
3	Choline chloride	Xylitol	1：1
4	Choline chloride	Lactic acid	1：2
5	Choline chloride	Citric acid	2：1
6	Betaine	1,2-Propylene glycol	1：2
7	Betaine	Urea	1：2
8	Betaine	Lactic acid	1：2
9	Betaine	Citric acid	2：1

Table S8. Response surface test factors and levels.

Symbol	Independent variable		
	A（%）	B（min）	C（℃）
-1	10	20	45
0	20	30	55
1	30	40	65

Table S9. Elution gradients for anion-exchange chromatography.

Time（min）	Mobile phase A（%）	Mobile phase B（%）	Mobile phase C（%）
0	95	5	0
26	85	5	10
42	85	5	10
42.1	60	0	40
52	60	40	0
52.1	95	5	0
60	95	5	0

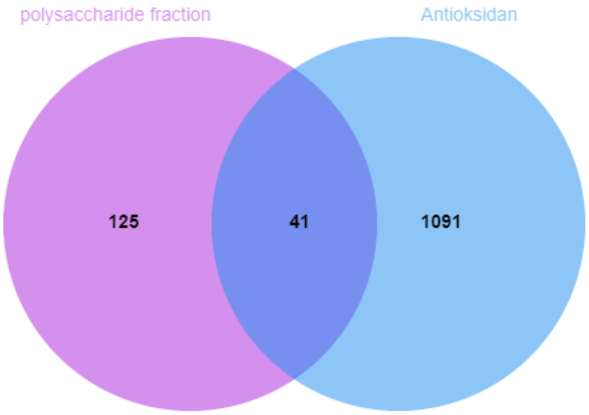

Figure S1. Venn diagram of drug targets and antioxidant targets.

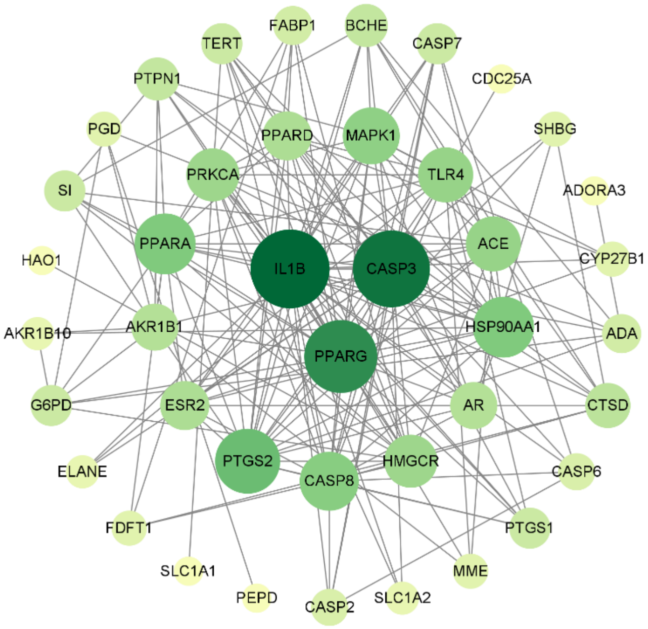

Figure S2. Visual PPI network diagram of antioxidant for Radix *Peucedani* polysaccharides.

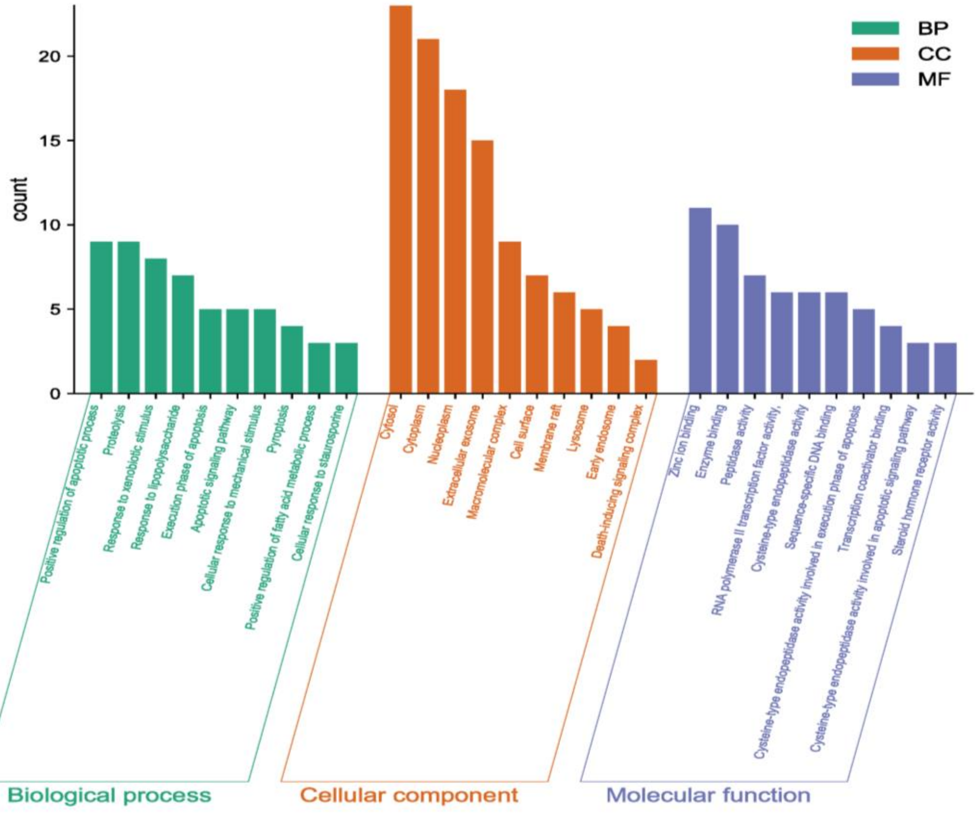

Figure S3. Analysis diagram of GO functional enrichment.

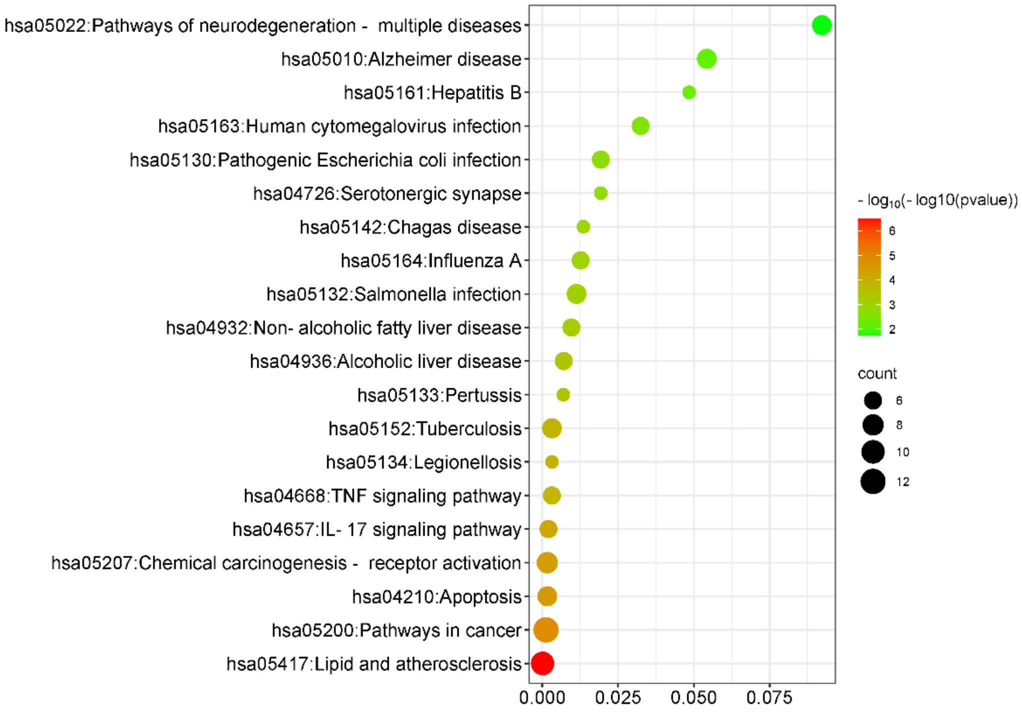

Figure S4. Enrichment analytic diagram of KEGG pathway.

Disclaimer/Publisher's Note: The statements, opinions and data contained in all publications are solely those of the individual author(s) and contributor(s) and not of MDPI and/or the editor(s). MDPI and/or the editor(s) disclaim responsibility for any injury to people or property resulting from any ideas, methods, instructions or products referred to in the content.
